# Comparative Effectiveness of Darunavir 1,200 mg Daily and Approved Dosing Strategies for Protease Inhibitor-Experienced Patients

**DOI:** 10.1155/2013/687176

**Published:** 2013-07-17

**Authors:** James M. Mikula, Chiu-Bin Hsiao, Joshua R. Sawyer, Qing Ma, Gene D. Morse

**Affiliations:** ^1^Immunodeficiency Services, Erie County Medical Center, 462 Grider Street, Buffalo, NY 14215, USA; ^2^Department of Pharmacy Practice, School of Pharmacy and Pharmaceutical Sciences, University at Buffalo, 285 Kapoor Hall, Buffalo, NY 14214, USA; ^3^Translational Pharmacology Research Core, New York State Center of Excellence in Bioinformatics and Life Sciences, 701 Ellicott Street, Buffalo, NY 14023, USA; ^4^Infectious Disease Division, Department of Medicine, University at Buffalo, The State University of New York, Buffalo, NY 14214, USA

## Abstract

*Background.* HIV protease inhibitors exhibit concentration-dependent viral inhibition. Higher once daily doses of darunavir boosted with ritonavir (DRV/r) may achieve viral suppression in place of twice daily dosing. International antiretroviral adherence guidelines recommend once daily regimens whenever possible. We present data on virologic suppression achieved with DRV 1,200 mg and ritonavir 100 mg once daily compared to approved DRV regimens. *Methods.* This retrospective observational study included all patients treated with DRV after documented use of another protease inhibitor at an urban immunodeficiency clinic. Data collection from inception of DRV use in August 2006 through March 2012 included patient demographics, viral loads, CD4+ cell counts, and resistance test results. The primary outcome of virologic suppression was defined as <50 copies/mL at 24 weeks. Differences in baseline characteristics and virologic outcomes across dosing groups were analyzed via one-way analysis of variance. *Results.* One hundred and thirty-five patients were included in the ITT analysis. Most patients had no known DRV RAMs at baseline. Virologic suppression rate was not different among treatment groups: 53.6% of patients on 1,200 mg daily, 52.3% on 600 mg twice daily, and 42.9% on 800 mg daily (*P* = 0.568). *Conclusions.* Darunavir 1,200 mg daily should be investigated for use in protease inhibitor-experienced patients.

Darunavir 600 mg with 100 mg of ritonavir (DRV/r) dosed twice daily is currently approved in treatment-experienced patients as part of combination antiretroviral therapy (cART) which effectively suppresses HIV in this patient population [1]. DRV/r 800 mg daily is approved for patients lacking any DRV resistance associated mutations (RAMs), including treatment-naïve patients [2]. DRV has a high genetic barrier to resistance enabling successful treatment even in the presence of multiple DRV RAMs [3]. The expansion of the antiretroviral armamentarium in recent years has led to ever more effective DRV/r-based cART against multiclass resistant HIV-1 [4, 5].

Treatment-experienced patients often have failed previous regimens due to poor adherence, adverse drug events, and the development of drug resistance. Therefore, the ideal regimen for treatment-experienced patients is well-tolerated, efficacious against drug-resistant virus, and convenient [6, 7]. Added emphasis has recently been placed on selecting once daily antiretroviral regimens for all patients to promote treatment adherence [7]. The regimen should suppress viremia below the lower limit of detection for the most sensitive assay available [6]. The protease inhibitor dose-response curve may allow greater virologic suppression with higher doses [8]. Inhibitory quotients, a ratio of drug concentration to a measure of drug susceptibility, have predicted protease inhibitor efficacy better than pharmacokinetic or resistance data alone [9–11]. DRV/r 1,200 mg daily results in drug exposure that is intermediate between the two currently approved dosing regimens [12]. This dose may strike a balance between achieving DRV exposure sufficient to overcome drug resistance while limiting ritonavir exposure and promoting adherence; however, published efficacy and safety data have been limited to case reports and pharmacokinetic studies [13, 14]. The objective of this study is to compare the effectiveness of DRV/r 1,200 mg once daily to currently approved DRV/r doses in protease inhibitor-experienced patients.

This retrospective cohort study screened all patients at our adult immunodeficiency clinic prescribed DRV since its initial use in August 2006 through March 2012. All patients with documented use of a protease inhibitor and subsequent therapy with DRV/r 1,200 mg daily, 600 mg twice daily, and 800 mg daily were included. Subjects who began DRV/r therapy <24 weeks before the study end date were excluded. The baseline and demographic data retrieved from the electronic medical record were age, sex, race/ethnicity, height, weight, start date of DRV/r therapy, CD4+ cell counts, viral loads (Roche COBAS AmpliPrep/Taqman HIV-1 test v1 and v2.0), and the number of concomitantly prescribed antiretrovirals. Protease inhibitor mutations were compiled from historical and on-treatment resistance tests (TruGene HIV-1; VircoType HIV-1). DRV RAMs were identified consistent with product labeling as determined from previous analyses [15, 16].

Viral load at 24 weeks (±12 weeks) was evaluated for the primary endpoint of viral suppression (defined as <50 copies HIV RNA per mL) for the intention to treat-time to loss of virologic response (ITT-TLOVR) algorithm. Discontinuation of DRV prior to 12 weeks of therapy or the absence of a viral load in the 12–36 weeks window was considered treatment failure. Two viral loads >50 copies/mL after viral suppression also were identified as treatment failures. The secondary endpoint utilized the same ITT-TLOVR algorithm to determine the rate of viral suppression at 48 weeks (±12 weeks). One-way analysis of variance was used to determine the significance of differences in baseline factors and results.

One hundred and eighty treatment-experienced patients started DRV/r of which 143 had previously documented use of another protease inhibitor. Of these, 135 subjects initiated DRV/r ≥24 weeks before the study end date and were included in the ITT analysis. Baseline patient data including resistance are presented in Table 1. Most patients had no documented resistance and <15% had 2 or more DRV RAMs. The 600 mg twice daily group trended toward higher baseline viral loads (*P* = 0.197), lower CD4+ cell counts (*P* = 0.003), and a larger share of Caucasian and Hispanic subjects versus patients of African descent (*P* = 0.198).

A similar percentage of patients treated with 1,200 mg daily and 600 mg twice daily reached the primary endpoint of a suppressed viral load at 24 weeks, with a statistically insignificant trend toward viral suppression over the 800 mg daily group (53.6%, 52.3%, and 42.9%, resp., *P* = 0.568). This difference was equalized by 48 weeks (45.8%, 49.2%, and 48.5%, *P* = 0.960). Of all subjects, 60.7% had their viral load suppressed to <200 copies/mL at 24 weeks (71.4% on 1,200 mg daily, 60.0% on 600 mg twice daily, and 52.8% on 800 mg daily). At 48 weeks, 54.1% of subjects were suppressed to <200 copies/mL (50.0%, 55.4%, and 54.5%). CD4+ cell counts increased slightly across groups (49, 25, and 17 cells/mL at 48 weeks), and all groups had an increase in CD4+ % (1.5%, 3.7%, and 3.8% at 48 weeks). No new DRV RAMs were documented during therapy.

The groups had important baseline differences. The greater drug resistance, lower CD4+ count, and larger cART regimen seen with DRV/r 600 mg twice daily indicate a population more difficult to achieve viral suppression. The lack of DRV RAMs detected could be due to a sometimes distant history of protease inhibitor use, including use predating implementation of resistance testing. This study does not assess the effect of DRV dose on adherence and tolerability, important predictors of effectiveness. The inherent adherence advantage of once daily dosing could be lost to poorer tolerability of a larger DRV dose taken at a single time. Prospective adherence surveys and therapeutic drug monitoring could establish pharmacokinetic differences between DRV doses, especially since the DRV 1,200 mg dose has only been studied in HIV-negative healthy volunteers [12].

Viral suppression rates appear low in this patient sample; however, effectiveness outcomes are less common and less favorable than clinical trial data which are affected by stricter exclusion criteria [4, 17]. Providers may select a darunavir regimen for patients at risk of nonadherence due to its high barrier to resistance. 

DRV/r 1,200 mg daily resulted in viral suppression in some protease inhibitor-experienced patients, though selection bias hinders comparisons with other dosing regimens in this study. A randomized controlled trial with safety, adherence, and therapeutic drug monitoring should be undertaken to elucidate the utility of this DRV dose.

## Figures and Tables

**Table 1:** Baseline characteristics and rate of viral suppression by darunavir dose.

	All patients	DRV 1,200 mg daily	DRV 600 mg twice daily	DRV 800 mg daily	*P* value
*N*	135	28	65	42	
Age, yr, median (IQR)	47 (43–52)	48 (42–51)	46 (42–51)	49 (44–54)	0.174
Race/ethnicity					0.198
African American (%)	48.1	60.7	36.9	57.1	
Caucasian (%)	33.3	25	38.5	31	
Hispanic (%)	17	14.3	21.5	11.9	
Other/unknown (%)	1.5	0	3.1	0	
Baseline viral load					0.197
<1,000 copies/mL (%)	43	45.2	33.8	45.2	
1,000–99,999 copies/mL (%)	40.7	40.5	46.2	40.5	
≥100,000 copies/mL (%)	16.3	14.3	20	14.3	
CD4+ cells/mL, mean (SD)	343.3	399.8 (355.1)	259.4 (229.6)	435.6 (283.5)	0.003
Concomitant ARVs, mean (SD)	2.2	1.86 (1.01)	2.55 (1.00)	1.93 (0.71)	<0.001
Baseline resistance					
Baseline resistance testing available (*n*)	109/135	20/28	57/65	32/42	
Total DRV RAMs at baseline	72	5	66	1	<0.001
Baseline DRV RAMs, median (range)	0 (0–4)	0 (0–3)	1 (0–4)	0 (0-1)	<0.001
Patients with ≥1 documented DRV RAM (%)	28.9	10.7	53.8	2.4	<0.001
Endpoints					
<50 copies/mL, 24 weeks (%)	49.6	53.6	52.3	42.9	0.568
<50 copies/mL, 48 weeks (%)	48.4	45.8	49.2	48.5	0.960
